# Grazing protozoa and the evolution of the *Escherichia coli* O157:H7 Shiga toxin-encoding prophage

**DOI:** 10.1098/rspb.2007.0245

**Published:** 2007-05-29

**Authors:** Karyn Meltz Steinberg, Bruce R Levin

**Affiliations:** 1 Department of Biology, Rollins Research Center, Emory UniversityAtlanta, GA 30322, USA; 2 Graduate Program in Population Biology, Ecology and Evolution, Emory UniversityAtlanta, GA 30322, USA

**Keywords:** Shiga toxin, *Escherichia coli* O157:H7, prophage, protozoa predation, *Tetrahymena pyriformis*

## Abstract

Humans play little role in the epidemiology of *Escherichia coli* O157:H7, a commensal bacterium of cattle. Why then does *E. coli* O157:H7 code for virulence determinants, like the Shiga toxins (Stxs), responsible for the morbidity and mortality of colonized humans? One possibility is that the virulence of these bacteria to humans is coincidental and these virulence factors evolved for and are maintained for other roles they play in the ecology of these bacteria. Here, we test the hypothesis that the carriage of the Stx-encoding prophage of *E. coli* O157:H7 increases the rate of survival of *E. coli* in the presence of grazing protozoa, *Tetrahymena pyriformis*. In the presence but not the absence of *Tetrahymena,* the carriage of the Stx-encoding prophage considerably augments the fitness of *E. coli* K-12 as well as clinical isolates of *E. coli* O157 by increasing the rate of survival of the bacteria in the food vacuoles of these ciliates. Grazing protozoa in the environment or natural host are likely to play a significant role in the ecology and maintenance of the Stx-encoding prophage of *E. coli* O157:H7 and may well contribute to the evolution of the virulence of these bacteria to colonize humans.

## 1. Introduction

*Escherichia coli* O157:H7 is responsible for a significant amount of human morbidity and mortality in the developed world (Besser *et al*. 1999; Rangel *et al*. 2005) and a recurrent source of economic privation for the food industry (DeWaal *et al*. 2006). However, *E. coli* O157:H7 is not a human pathogen in the sense that human-to-human transmission is rare and not sufficient to sustain populations of these bacteria indefinitely. Cattle and other ungulates, in which these enteric bacteria rarely cause disease, are considered to be the natural host of *E. coli* O157:H7 (Dean-Nystrom *et al*. 1998*a*; Hancock *et al*. 1998). Why then do *E. coli* O157:H7 and the prophage they carry code for toxins and other virulence factors that are responsible for morbidity and mortality of colonized humans?

A probable answer is that the Shiga toxins (Stx) and other factors responsible for the virulence of these bacteria in humans evolved and are maintained by selection for other roles they play in the ecology of these bacteria. In this interpretation, the virulence of *E. coli* O157:H7 in colonized humans is coincidental, an inappropriate or over response to these factors. But what other roles do the virulence determinants of *E. coli* O157:H7 play?

One possibility is that the virulence factors responsible for human disease facilitate *E. coli* O157's colonization, replication or transmission in their bovine hosts. There is evidence for this being the case. For example, *E. coli* O157 bear a pathogenicity island containing the *eae* locus, which promotes colonization in cattle via attaching and effacing lesions (Dean-Nystrom *et al*. 1998*b*; Cornick *et al*. 2002; Sheng *et al*. 2006). Moreover, bovine intestinal cells have receptors for the Stxs (Menge *et al*. 2004) expressed by these bacteria and, at least in a mouse model, Stx 2 promotes intestinal colonization (Robinson *et al*. 2006).

Another possibility is that some of the virulence factors enhance the survival of these bacteria by providing protection against predation by bactivorous protozoa, nematodes or other predators in the soil, water or the gastrointestinal tract of their bovine hosts. Bacteria, including pathogenic coliforms (King *et al*. 1988), are grazed upon by bactivorous protozoa, and this predation is considered to be important in shaping the structure of bacterial communities in planktonic as well as terrestrial ecosystems (Hahn & Hofle 2001; Jurgens & Matz 2002). If indeed predation by protozoa contributes to bacterial mortality, it would not be surprising at all that these bacteria have evolved ways to resist this predation. There are, in fact, a variety of mechanisms that bacteria use to either evade or survive protozoan predation (Matz & Kjelleberg 2005). There is also evidence for within- and between-species variation in the susceptibility of bacteria to protozoan predation. Some serotypes of *Salmonella enterica* are more resistant to predation by amoeba than others (Tezcan-Merdol *et al*. 2004; Wildschutte *et al*. 2004). *Salmonella enterica* serovar Thompson survive better than *Listeria monocytogenes* within the food vacuoles of *Tetrahymena pyriformis* (Brandl *et al*. 2005). In a recent review, it has been suggested that *E. coli* O157:H7 may have a survival advantage in the food vacuoles of these bactivorous ciliates (Brandl 2006).

Here, we present the results of experiments testing the hypothesis that the Stx-encoding prophage of *E. coli* O157 provides protection against predation by grazing protozoa. We demonstrate that in the presence but not the absence of *T. pyriformis*, *E. coli* K-12, as well as O157:H7 and O157:H−, that is lysogenic for this lambda-like phage has an advantage over non-lysogens. Our results suggest that part, but not all, of this protection is associated with Stx 2 (Stx2) and can be attributed to the prophage coding for this toxin, increasing the rate of survival of these bacteria in the food vacuoles of these ciliate predators.

## 2. Material and methods

### (a) Strains

All strains are listed in table 1. Strains include human clinical isolates from stool and laboratory-made lysogens created using the K-12 derivative, C600, lysogenized with Stx2-encoding prophages from 933W (CDC) and 3538/95 (Schmidt *et al*. 1999); these strains are called C600P and C600PT+, respectively, in this paper. Additionally, C600 was lysogenized with the 3538/95Δ*tox::cat* prophage in which the toxin genes have been deleted and a chloramphenicol resistance cassette inserted in its place (Schmidt *et al*. 1999), and this strain will be known as C600PT−. The identity of bacterial genotypes was confirmed by biotyping and with PCR markers, and that of phage genotypes was confirmed by restriction digest analysis of purified phage DNA (M. Reynolds 2000, unpublished data).

The C600 and C600P strains were transformed with a commercially available green fluorescent protein (*gfp*)-encoded plasmid (Clontech) and are referred to as C600*gfp* and C600P*gfp*, respectively. Transformation protocols can be found in Sambrook *et al*. (1989). Cultures were grown in Luria–Bertani (LB) broth with 5% ampicillin until reaching a density of 0.3 at 600 nm, at which point they were induced with 0.1 mM isopropyl β-d-1-thiogalactopyranoside (IPTG) and incubated for an additional 16 h at 37°C on a rotary shaker.

For all experiments, bacteria were grown in LB broth (with appropriate antibiotics) on a rotary shaker at 37°C. In mixed culture experiments, arabinose, lactose, chloramphenicol and nalidixic acid markers were used to distinguish toxin-positive from toxin-negative bacteria by selective plating on tetrazolium agar with arabinose or lactose, or LB agar with chloramphenicol (2.5%) or nalidixic acid (2.0%). Axenic cultures of *T. pyriformis* were obtained from Carolina Biological Supply Co. (Burlington, NC). *Tetrahymena pyriformis* is a freshwater and soil ciliate that commonly inhabits lakes and streams. These organisms will grow in axenic or defined medium and tolerate temperatures up to 22°C. For all experiments, *Tetrahymena* were grown in proteose peptone/tryptone media (5 g proteose peptone, 5 g tryptone and 0.2 g K_2_HPO_4_ in 1 l H_2_O, pH adjusted to 7.2) in a stationary culture flask to a density of 10^5^ cells ml^−1^ at 24–27°C.

### (b) Mixed culture experiments

Mixed cultures were maintained in the presence and absence of *T. pyriformis*. The cultures were maintained without agitation at room temperature, 22–27°C. Samples were taken every 24 h for 7 days, and the total cell densities and relative frequencies of prophage (or toxin)-positive and -negative bacteria were estimated by counting the number of colony-forming units (CFUs). *Tetrahymena* densities were estimated by counting the number of ciliates in 10 μl samples stained with 10 μl of Lugol's iodine using a Neubauer improved haemocytometer and an inverted microscope. Toxin production was measured using a commercially available EHEC ELISA kit (Meridian, Inc.).

### (c) Vacuole production and bacterial cell number per vacuole

To determine the number of vacuoles produced per *Tetrahymena* and measure the number of bacterial cells per vacuole, we used a modified version of the protocol described in Brandl *et al*. (2005). Briefly, stationary-phase bacteria from cultures of C600*gfp* and C600P*gfp* were washed twice, resuspended in sterile distilled water and serially diluted in sterile distilled water to 10^8^ cells ml^−1^. One millilitre of prophage-positive or prophage-negative bacteria was placed in sterile Eppendorf tubes, and 5×10^4^ *Tetrahymena* cells were added to the tubes. Tubes were incubated for 24 h at room temperature without shaking. At 12 and 24 h, samples were taken and bacterial densities were estimated from the CFU data. At 24 h, 10 μl aliquots were placed on a clean Polysine slide and viewed under an epifluorescent microscope. The numbers of green cells per vacuole were counted for 30 vacuoles from duplicate tubes. Bacterial viability within vacuoles was assessed using the Live/Dead *Bac*Light viability system (Molecular Probes, Eugene, OR).

### (d) Statistical methods

The *t*-tests assuming unequal variances were used to test the significance between the initial bacterial ratios and densities and the ratios and densities after day 3. This test was also used to compare the mean numbers of bacterial cells per vacuole.

## 3. Results

### (a) The Stx-encoding prophage of *E. coli* O157 augments the fitness of *E. coli* K-12 in the presence of *T. pyriformis*

An *E. coli* K-12 derivative, C600 (Ara+) and C600 (Ara−) lysogenized with a Stx2-encoding prophage, C600P, were separately grown overnight in LB broth, washed and resuspended in sterile distilled water to full volume. Equal frequencies of prophage-positive (C600P) and prophage-negative (C600) suspensions totalling 2 ml were added to the 5 ml wells of 12-well macrotitre plates and approximately 500 unstarved (10 μl) *T. pyriformis* from axenic cultures were added to each experimental well for total densities of approximately 250 ciliates and 10^9^ bacteria ml^−1^. These plates were maintained at room temperature (approx. 22°C) without agitation. At 24 h intervals, the densities of C600P and C600 were estimated from the CFU data on tetrazolium arabinose agar and that of *Tetrahymena* microscopically with a haemocytometer.

As shown in figure 1*a*, there is no change in the ratio of C600P to C600 in the *Tetrahymena*-free controls but a nearly two order of magnitude increase in the ratio of C600P to C600 in the cultures with these grazing ciliates (*p*<0.002). The C600P/C600 ratio (figure 1*a*) increases when the density of *Tetrahymena* increases and levels off when there is no longer a change in the density of *Tetrahymena*.

### (b) Predation dynamics

On first consideration, it may seem curious that the *Tetrahymena* population appears to stop growing while there are still substantial numbers of bacteria, approximately 10^6^ ml^−1^. This had been observed and explained by Watson and colleagues in their studies with *T. pyriformis* and washed *E. coli* from chemostats (Watson *et al*. 1981). These authors suggest that there is a threshold density of approximately 10^5^ bacteria ml^−1^ below which *Tetrahymena* no longer feed on the *E. coli*. To ascertain whether this threshold also exists under the conditions of our experiments, we diluted washed stationary-phase C600 cultures to 10^5^ cells ml^−1^ and added 250 *Tetrahymena* ml^−1^. The density of *E. coli* remained at 10^5^ cells ml^−1^ and there was no growth of the protozoa (data not shown). Moreover, if we consider the initial density of bacteria in these experiments and the maximum density *Tetrahymena* achieve, approximately 10^9^ and 10^5^ cells ml^−1^ (see figure 1*b*), our data are also consistent with that of Watson and colleagues (Watson *et al*. 1981); it requires consumption of approximately 10^4^ *E. coli* to produce a single *Tetrahymena*.

### (c) The Stx-encoding prophage augments the fitness of *E. coli* O157:H7 and *E. coli* O157:H− in the presence of *Tetrahymena*

To ascertain whether the advantage of *E. coli* lysogenic for the Stx-encoding prophage in the presence of *Tetrahymena* obtains for *E. coli* O157 lysogens as well as *E. coli* K-12 lysogens, we performed these pairwise mixed culture experiments with C600 and clinical isolates of *E. coli* O157:H7 and *E. coli* O157:H−. In figure 2, we plot the ratio of *E. coli* O157 and C600 at the start of these experiments and after 3 days in the presence and absence of *Tetrahymena*.

When *E. coli* O157 carry prophage that codes for Stx1 and Stx2 (figure 2*a*), the results are consistent with those of the analogous experiments with C600P and C600. In the presence but not the absence of *Tetrahymena*, these lysogenic clinical isolates have a fitness advantage over the C600. The results of the experiments with clinical *E. coli* O157 isolates that carry prophage coding for Stx1 but not Stx2 or no prophage are more ambiguous (figure 2*b*,*c*). One clinical isolate of *E. coli* O157:H−, 85-3476 (*stx1*), had an advantage over C600 in the presence but not the absence of *Tetrahymena*. However, two *E. coli* O157:H7 strains, 89-3360 (*stx1*) and 00-3150 (*stx*−), had no apparent advantage in the presence or absence of *Tetrahymena*.

### (d) Role of the stx genes in augmenting the fitness of *E. coli* in the presence of *Tetrahymena*

The results of the above-described experiments with clinical strains of *E. coli* O157 support a role for *stx2* in augmenting the fitness of *E. coli* carrying prophage with this gene in the presence of *Tetrahymena*. Further evidence for this comes from mixed culture experiments we performed with *E. coli* carrying prophage in which the *stx2* locus was inactivated.

Four *E. coli* lysogens were used in these experiments: (i) C600PT+ which carry a prophage with a functional *stx2*, (ii) C600PT− which carry the same prophage as C600PT+, but in which the *stx2* locus was replaced with a chloramphenicol resistance cassette (Schmidt *et al*. 1999), (iii) P2T+, an *E. coli* O157:H7 strain which carry a prophage with a functional *stx2* locus, and (iv) P2T−, the above *E. coli* O157:H7 which carry the same prophage as P2T+, but in which the *stx2* locus is deleted (Gunzer *et al*. 1998). These Stx+ and Stx− lysogens were mixed with C600 (figure 3). We also performed these pairwise mixed culture experiments with mixtures of otherwise isogenic Stx+ and Stx− lysogens (figure 4).

For both the C600 and *E. coli* O157:H7 constructs, bacteria bearing prophage with a functional *stx2* gene have an advantage over C600 in the presence but not the absence of *Tetrahymena* (figure 3*a*,*b*). The results of experiments with lysogens bearing prophage with inactivated *stx2* loci suggest that even without a functional Stx2, this prophage increases the fitness of *E. coli* in the presence but not the absence of *Tetrahymena* (figure 3*c*,*d*). The magnitude of the advantage appears to be greater when the *stx2* locus is functional. Additional support for this interpretation comes from experiments where the *stx*-positive and *stx*-negative lysogens are mixed with each other. In the presence but not the absence of *Tetrahymena*, the *stx2*-positive bacteria have a fitness advantage over the *stx2*-negative bacteria (figure 4).

### (e) The Stx-encoding prophage increases the survival of *E. coli* in the food vacuoles of *Tetrahymena*

How do *Tetrahymena* select for prophage-bearing *E. coli*? One possibility is that the carriage of the prophage increases the rate of survival of *E. coli* ingested by *Tetrahymena* (Brandl *et al*. 2005) relative to bacteria not carrying this element. During grazing, *Tetrahymena* fill organelles known as food vacuoles with bacteria which are lysed and digested over a period of time. Bacteria-filled food vacuoles are also released into the environment (King *et al*. 1988; Schlimme *et al*. 1995). In accordance with this hypothesis, bacteria with higher rates of survival in food vacuoles would be more likely to be recovered as CFUs by plating either vacuoles or bacteria released from vacuoles, than bacteria with lower rates of survival.

To test this hypothesis, we labelled C600 and C600P with *gfp* (pEGFP, Clontech), C600*gfp* and C600P*gfp*, respectively. To washed, overnight, single clone cultures of C600*gfp* and C600P*gfp*, we added approximately 5×10^4^ *Tetrahymena* and incubated the suspensions at room temperature for 24 h. At 12 and 24 h, we estimated densities of bacteria by plating (CFU) and counting the number of bacteria per food vacuole using an epifluorescent microscope. To test the viability of the bacteria in the food vacuoles, we used the Live/Dead *Bac*Light viability system (Molecular Probes, Eugene, OR).

The results of our plating experiments suggest that the colonies on the plates arise from single cells rather than vacuoles containing multiple bacteria. The colonies on the tetrazolium arabinose agar did not contain Ara− and Ara+ sectors and when restreaked on this indicator agar, the colonies produced were monomorphic Ara− or Ara+. As estimated from either colony count data or number of fluorescing bacteria per vesicle, there are more surviving prophage-bearing *E. coli* in the presence of *Tetrahymena* than prophage-free bacteria (figure 5*a*,*b*). The survival of the bacteria within the food vacuoles can be seen from confocal micrographs of *Tetrahymena* in figure 5*c*,*d*. In figure 5*c*, where C600 carries the prophage, there are an abundance of viable cells within the food vesicles. This is clearly not the case when C600 does not carry the prophage (figure 5*d*). In the absence of *Tetrahymena*, there is no difference in the rate of survival of the bacteria with the *gfp* with and without the prophage (data not shown).

Additional evidence for the enhanced survival of C600P within the food vacuoles was obtained from the results of experiments using the Live/Dead *Bac*Light viability assay. As determined by this protocol, which measures bacterial membrane integrity, a substantial fraction of C600P cells in the vacuoles were viable; they did not take up propidium iodide. On the other hand, few, if any, C600 cells failed to take up propidium iodide (data not shown).

## 4. Discussion

The results of our *in vitro* experiments with mixed cultures of *E. coli* bearing and not bearing the Stx-encoding prophage of *E. coli* O157:H7 are consistent with the hypothesis that predation by protozoa favours lysogens for this prophage. In the presence but not the absence of *T. pyriformis*, the carriage of the Stx-encoding prophage augments the fitness of both the laboratory strain *E. coli* K-12 (C600) and the clinical isolates of *E. coli* O157:H7 and *E. coli* O157:H−.

Our experiments with *E. coli* with prophage carrying non-functional *stx* loci suggest that the advantage of these lysogens in the presence of grazing protozoa can, to some extent, but not entirely, be attributed to Stx2. In the presence but not the absence of *Tetrahymena*, all of the clinical isolates of *E. coli* O157 carrying prophage with functional *stx2* loci had an advantage over C600 non-lysogens as well as otherwise isogenic cells with non-functional *stx2* alleles. On the other hand, one clinical isolate bearing *stx1* but not *stx2* also had an advantage over C600 in the presence but not the absence of *Tetrahymena*.

Our results indicated that some, if not all, of the advantage of *E. coli* with the Stx-encoding prophage in the presence of *Tetrahymena* can be attributed to the prophage increasing the rate of the survival of these bacteria within intact food vacuoles. While it has been suggested that build-up of toxin in the food vacuoles leads to their expulsion by the protozoa before all the bacteria are digested (Matz & Kjelleberg 2005), toxin-mediated expulsion alone would not account for the observed advantage of the prophage-bearing cells. In these mixed cultures, the vacuoles would contain both lysogens and non-lysogens for this phage. If these vacuoles are expelled before the bacteria are digested or bacteria with and without prophage survive equally well in these vacuoles, there would be no advantage associated with the carriage of these prophages. On the other hand, owing to their greater rate of survival in the vacuoles before expulsion, the relative frequency of Stx-prophage-bearing bacteria in the extra-ciliate environment at large would continue to increase as long as the ciliates grazed on these bacteria.

Under what conditions, in what habitats and to what extent predation by protozoa contributes to the persistence of Stx-encoding phages in *E. coli* are, at this time, unanswered questions. Is this protozoa-mediated selection for *E. coli* bearing Stx-encoding prophage occurring in the external environment, water or soil, or in the gastrointestinal tract of colonized bovines? The density of *E. coli* in the rumen of cattle, which has been estimated to be less than 10^5^ cells g^−1^ (Russell *et al*. 2000), may be too low for the protozoa to be effective predators and thereby to select for bacteria carrying Stx-encoding prophage. If, however, the total density of bacteria in the rumen is sufficient to maintain a substantial population of grazing ciliates and possibly other even if *E. coli* is a small minority population, selection could still favour *E. coli* carrying Stx-encoding prophage. This can be seen with the simple mathematical model where we consider three populations of bacteria (Appendix A).

Bactivorous protozoa are present in the lower colon (Coleman 1964), and that too may be a habitat for protozoa-mediated selection favouring *E. coli* bearing Stx-encoding prophage. The bovine rectal mucosa is the primary site of *E. coli* O157:H7 colonization (Naylor *et al*. 2003). In grain-fed cattle, the densities of *E. coli* in the colon have been reported to be of the order of 10^8^ cells ml^−1^ (Russell *et al*. 2000), and at *E. coli* densities of this magnitude, there is evidence that gastrointestinal protozoa take up these bacteria at a high rate (Coleman 1964). As mentioned above and illustrated in Appendix A, even if the density of *E. coli* O157:H7 in the intestine is too low of a density to sustain a population of grazing protozoa by itself, if the majority population of bacteria consumed by these protozoa is dense enough, selection mediated by predation would still favour the carriage of the Stx-encoding prophage in the *E. coli* O157:H7 minority.

The results of our *in vitro* experiments as well as other studies of bacterial predation by grazing protozoa point to the need to more extensively evaluate the contribution of this predation to the maintenance of Stx-encoding prophage in the ecology of *E. coli* O157:H7 and possibly *E. coli* O157:H7 itself. Mixed culture experiments similar to those reported here with prophage-bearing and prophage-free *E. coli* O157 could be performed *in situ* as well as *in vitro* with naturally occurring protozoa from the gastrointestinal tracts of cattle and other ungulates. These protozoa include species from genera *Eudiplodinium*, *Metadinium*, *Polyplastron*, *Isotricha*, *Entodinium* and *Diplodinium* (Rasmussen *et al*. 2005), which are all bactivorous ciliates like *Tetrahymena*. These experiments can also be performed in the soil and water in areas where cattle graze and where the faeces of cattle are used for fertilizer or are a probable source of contamination.

It is conceivable that the increased capacity to survive in the food vacuoles of protozoa enhances the term of survival of *E. coli* O157:H7 in the environment and thereby the likelihood of their transmission to humans. A number of studies have shown that bacteria ingested by protozoa are more resistant to disinfectants and biocides than free bacteria (Watson *et al*. 1981; Barker *et al*. 1992; Brandl *et al*. 2005). Could this be the case for the *E. coli* O157:H7 contaminants of bean sprouts, lettuce and spinach? Protozoa could also play a role in the transmission of *E. coli* O157:H7 between cattle and thereby the persistence of these bacteria in time and space. At any given time, the overall prevalence of *E. coli* O157:H7 on a single farm may be too low for a few individual colonized cattle shedding bacteria (Matthews *et al*. 2006) serving as a source for the persistence of these bacteria in the herd at large. However, anything that promotes the survival of *E. coli* O157:H7 in the extra-host environment could also contribute to the maintenance of these bacteria in the herd as well as their spread to other farms (Wetzel & LeJeune 2006). Moreover, if *E. coli* O157:H7 is like *Legionella pneumophila* and *S. enterica*, their carriage in protozoa may also make them more virulent to humans than when they are ingested as free bacteria (Cirillo *et al*. 1994; Rasmussen *et al*. 2005). Finally, it is possible that ingestion by and survival in these unicellular eukaryotes may be the selective force responsible for the evolution of the toxins and other prophage-encoded virulence factors of *E. coli* O157:H7. In this interpretation, protozoa are acting like what Barker and Brown refer to as ‘Trojan horses’ (Barker & Brown 1994).

To be sure, these extensions of the results of this *in vitro* study are, at this stage, no more than speculations. They are however speculations (hypotheses) that can be tested experimentally. We believe that these results and hypotheses have the dual virtues of being interesting to ecology, population and evolutionary biology as academic subjects and, at the same time, relevant to our understanding of the epidemiology of an infectious disease and the development of procedures for its control.

## Figures and Tables

**Figure 1:**
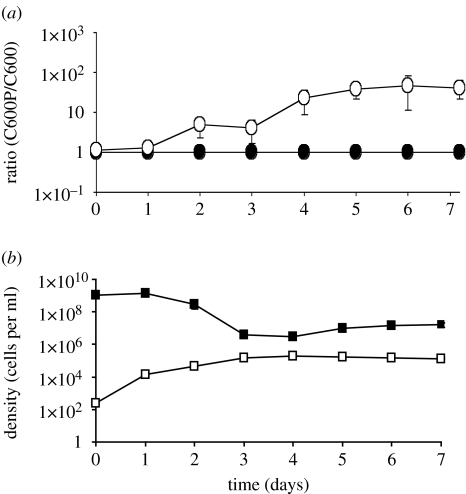
Mixtures of *E. coli* C600 with and without the Stx2-encoding prophage, C600 and C600P, respectively, in the presence and absence of *Tetrahymena*. Means ±s.e. for two control (*Tetrahymena*-free) and six experimental (with *Tetrahymena*) cultures. (*a*) Change in ratio of C600P/C600 in the presence and absence of *Tetrahymena*. Open circles, with *Tetrahymena*; filled circles, without *Tetrahymena*. (*b*) Change in the total densities of bacteria and *Tetrahymena* in the culture shown in (*a*). Filled squares, total bacteria; open squares, *Tetrahymena*.

**Figure 2:**
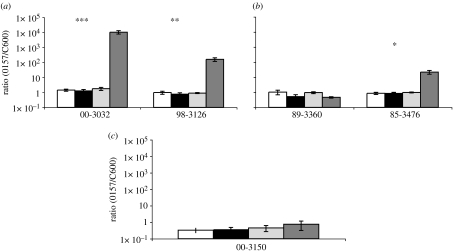
Mixtures of clinical isolates of *E. coli* O157:H7 or *E. coli* O157:H− and C600 in the presence and absence of *Tetrahymena*. Ratio of O157 to C600 initially and after 3 days with and without *Tetrahymena*, means ±s.e. (*a*) Clinical isolates with Stx1- and Stx2-encoding prophage. (*b*) Clinical isolates with Stx1-encoding prophage. (*c*) Clinical isolates with no Stx-encoding prophage. For each experiment, there were two control and six experimental cultures (^*^p<0.05, ^**^p<0.005 and ^***^p<0.0005). White bars, initial without *Tetrahymena*; black bars, after 3 days without *Tetrahymena*; light grey bars, initial with *Tetrahymena*; dark grey bars, after 3 days with *Tetrahymena*.

**Figure 3:**
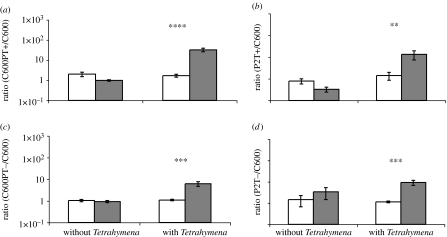
Mixtures of lysogens and non-lysogens in the presence and absence of *Tetrahymena* initially and after 3 days, means ±s.e. (*a*) Ratio of C600 with an Stx2-encoding prophage, C600PT+, and C600. (*b*) Ratio of *E. coli* O157:H7 with an Stx2-encoding prophage, P2T+, and C600. (*c*) Ratio of C600 with the toxin-negative construct of the Stx2-encoding prophage, C600PT−, and C600. (*d*) Ratio of the toxin-negative *E. coli* O157:H7 construct of the Stx2-encoding prophage, P2T−, and C600. In all experiments, there were three control and nine experimental cultures (^**^p<0.005, ^***^p<0.0005 and ^****^p<0.00005). White bars, initial; grey bars, after 3 days.

**Figure 4:**
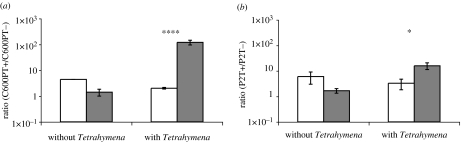
Mixtures of *stx2*-positive strains and isogenic *stx2*-negative constructs in the presence and absence of *Tetrahymena* initially and after 3 days, means ±s.e. (*a*) Ratio of C600PT+ and C600PT−. (*b*) Ratio of P2T+ and P2T−. For each experiment, there were two control and six experimental cultures (^*^p<0.05 and ^****^p<0.00005). White bars, initial; grey bars, after 3 days.

**Figure 5:**
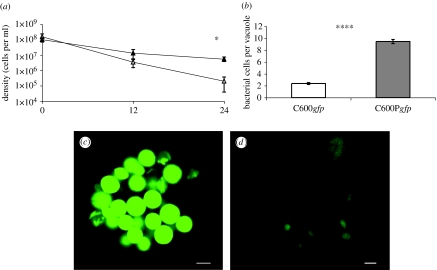
C600*gfp* (prophage negative) and C600P*gfp* (prophage positive) exposed to high density of *Tetrahymena*. (*a*) Estimated bacterial densities (CFU data) at 12 and 24 h, C600P*gfp* (closed triangles) and C600*gfp* (open triangles). (*b*) Number of viable C600*gfp* and C600P*gfp* cells per food vacuole, direct counts of fluorescing cells (^*^p<0.05 and ^****^p<0.00005). (*c*) C600P*gfp* in food vacuoles. (*d*) C600*gfp* in food vacuoles. Scale bar, 5 μm.

**Figure 6:**
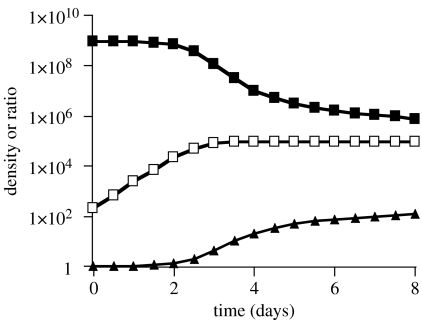
Simulation result changes in the total density of bacteria and protozoa and the ratio of prophage-bearing (Tox+) and prophage-free (Tox−) *E. coli* 0157:H7. Parameters *γ*_*B*_=2×10^−6^, *γ*_*M*_=2×10^−6^ and *γ*_*T*_=5×10^−7^; *e*=10^−4^, *k*=10^−7^. The initial densities of the populations were *B*=109, *M*=*T*=5×10^4^ and *P*=2×10^2^ cells ml^−1^. Open squares, density of *Tetrahymena*; filled squares, density of bacteria; filled triangles, ratio (Tox+/Tox−).

**Table 1:** List of *E. coli* strains and constructs used in this investigation.

strain designation	strain	Shiga toxin	motility	marker^a^	source
C600	C600	—	—	Lac-	this study
C600P	C600Φ933W	2	—	Ara-	this study
C600PT+	C600Φ3538/95	2	—		this study
C600PT−	C600Φ3538/95Δ*tox::cat*	—	—	Cam^R^	this study
C600g*fp*	C600P*gfp*	—	—	Ara-/Amp^R^	this study
C600P*gfp*	C600Φ933WP*gfp*	2	—	Ara-/Amp^R^	this study
PT+	O157:H7 3538/95	2	+		Schmidt *et al*. (1999)
PT−	O157:H7 3538/95Δ*tox::cat*	—	+	Cam^R^	Schmidt *et al*. (1999)
P2T+	O157:H7 86-24	2	+		Gunzer *et al*. (1998)
P2T−	O157:H7 TUV86-2	—	+	Nal^R^	Gunzer *et al*. (1998)
00-3032	O157:H7 00-3032	1,2	+		CDC
98-3126	O157:H− 98-3126	1,2	—		CDC
00-3150	O157:H7 00-3150	—	+		CDC
89-3360	O157:H7 89-3360	1	+		CDC
85-3476	O157:H− 85-3476	1	—		CDC

a Used for sampling.

## Appendix A

In our article, we suggest that even if *E. coli* O157:H7 is a minority population of insufficient density to support a grazing population of protozoa, protozoa-mediated selection could still favour the ascent of Stx-encoding *E. coli* O157:H7. The necessary condition for this is that the dominant population(s) of bacteria, be them *E. coli* or not, are grazed by these protozoa and are sufficiently dense for these protozoa to grow to substantial densities. Here, we use a simple mathematical model and numerical analysis of its properties to illustrate that, at least in theory, this would obtain.

In this model, we assume that there is one population of protozoa of density *P* and three populations of bacteria of densities and designations *B*, *M* and *T* cells ml^−1^. The *B* population of bacteria is consumed by the protozoa but is not *E. coli* O157. The *M* and *T* populations are, respectively, prophage-free and prophage-bearing *E. coli* O157:H7, Tox− and Tox+. We assume that bacteria and protozoa encounter each other at random at rates proportional to their densities, the standard mass action assumption of predator–prey models (e.g. Wilson & Bossart 1971). To account for the observation that *Tetrahymena* stop feeding when the density of bacteria becomes too low (Watson *et al*. 1981), unlike standard predator–prey models, we assume that the rate at which bacteria are consumed upon contact with the protozoa increases monotonically with the density of the total population of prey (bacteria). For the consumption dynamics, we assume hyperbolic function of the sort employed by Monod (1949). The constants *γ*_*B*_, *γ*_*M*_ and *γ*_*T*_ are the maximum rate parameters of consumption of the *B*, *M* and *T* bacterial population. The parameter *k* is the density of bacteria when the rate at which they are consumed upon contact with the protozoa is half its maximum value. We assume that the bacteria do not replicate and die only through predation by the protozoa. The protozoa do not die over the course of the experiment and only replicate by the consumption of the bacteria. A protozoan requires *e* bacteria to divide, the conversion efficiency (Stewart & Levin 1984). With these definitions and assumptions, the rates of change in the density of bacteria and protozoa are given by the following set of differential equations:

$$\begin{array}{c} \frac{\text{d}B}{\text{d}t}=-\gamma_{B}\phi (N)BP\\ \frac{\text{d}M}{\text{d}t}=-\gamma_{M}\phi (N)MP\\ \frac{\text{d}T}{\text{d}t}=-\gamma_{T}\phi (N)TP\\ \frac{\text{d}/P}{\text{d}T}=P\phi (N)(\gamma_{B}B+\gamma_{M}M+\gamma_{T}T)e, \end{array}$$

where *N*=*B*+*M*+*T* and *ϕ*(*N*)=*N/*(*N*+*k*)

For our illustration, we use a numerical solution to these equations, a computer simulation, programed in Berkeley Madonna. Copies of this program are available on www.eclf.com. Save for the conversion efficiency *e*, which has been estimated to be approximately 10^−4^ bacteria (Watson *et al*. 1981) and the present study, i.e. it takes approximately 10^4^ bacteria to produce a single protozoon, the other parameters were chosen to provide an approximate fit of *Tetrahymena*–*E. coli* observations in figure 1 of our report. In these simulations, we assume a delay of 4 h between the time the bacteria are consumed and the time the protozoa are produced.

With the chosen parameter values and initial densities of bacteria and protozoa, as with the real data in figure 1*a*,*b* in our article, the ratio of Tox+/Tox− increases when the density of protozoa increases. As the total density of the bacteria declines, the rate of ascent of the protozoa population declines and, within a few days, the density of both bacteria and protozoa levels off (figure 6).
